# MiR-452 Regulates C2C12 Myoblast Proliferation and Differentiation *via* Targeting *ANGPT1*

**DOI:** 10.3389/fgene.2021.640807

**Published:** 2021-03-12

**Authors:** Lingzhi Yang, Qi Qi, Jian Wang, Chengchuang Song, Yanhong Wang, Xi Chen, Hong Chen, Chunlei Zhang, Linyong Hu, Xingtang Fang

**Affiliations:** ^1^Institute of Cellular and Molecular Biology, Jiangsu Normal University, Xuzhou, China; ^2^Key Laboratory of Adaptation and Evolution of Plateau Biota, Northwest Institute of Plateau Biology, Chinese Academy of Sciences, Xining, China

**Keywords:** miR-452, C2C12 myoblasts, proliferation, differentiation, *ANGPT1*

## Abstract

microRNAs are a kind of endogenous, non-coding, single-strand small RNA. They have been reported as an important regulatory factor in skeletal myogenesis. In this study, miR-452 was selected from RNA high-throughput sequencing data to explore its regulatory role in myogenesis. Functionally, miR-452 overexpression could promote C2C12 myoblast proliferation while inhibiting myogenic differentiation. On the contrary, inhibition of miR-452 could suppress C2C12 myoblast proliferation but accelerate myogenic differentiation. Bioinformatics analysis and dual luciferase report assays showed that *Angiopoietin 1* (*ANGPT1*), *RB1*, and *CACNB4* were the potential target genes of miR-452. To further confirm the target relationship between *ANGPT1*, *RB1*, and *CACNB4* with miR-452, the mRNA level and protein level of these genes were detected by using RT-qPCR and Western blot, respectively. Result analysis indicated that *ANGPT1* was a target gene of miR-452. In addition, knockdown of *ANGPT1* could obviously promote C2C12 myoblast proliferation but block their differentiation. In summary, these results demonstrated that miR-452 promoted C2C12 myoblast proliferation and inhibited their differentiation *via* targeting *ANGPT1*.

## Introduction

Skeletal muscle development is an orchestral process. During the embryonic stage, myogenic progenitor cells undergo directional differentiation to form myoblasts, and after further proliferation, differentiation, and fusion, which eventually form multinuclear myotubes. The development process is regulated by myogenic regulatory factors (MRFs) and members of the myocyte enhancer factor 2 (MEF2) family, as well as by some non-coding RNAs likely microRNAs (miRNAs) (Yusuf and Brand-Saberi, 2012).

MiRNAs are a kind of short non-coding RNAs of approximately 22 nucleotides in length, which are involved in posttranscriptional regulation of gene expression (Ambros, 2004) and have been implicated in many biological processes, including skeletal muscle development (Mok et al., 2017). According to the expression feature of the miRNAs in skeletal muscle, they were classified into two categories, muscle-specific miRNAs or muscle-non-specific miRNAs. These muscle-specific miRNAs include miR-1, miR-133, miR-206, miR-486, miR-208, and miR-499 (Nie et al., 2015). miR-1, miR-133, and miR-206 promote the differentiation of muscle-derived progenitor cells through downregulating Baf60a and Baf60c (Goljanek-Whysall et al., 2014). MiR-486 could inhibit myoblast proliferation while promoting differentiation by targeting Pax7 (Dey et al., 2011). miR-208 and miR-499 belong to the same miRNA family, which could participate in the regulation of muscle fiber-type transformation and energy metabolism (Gan et al., 2013). In addition, muscle-non-specific miRNAs like miR-192 (Zhao et al., 2016), miR-17-92 (Qiu et al., 2016), miR-30-5p (Zhang et al., 2016), and miR-660 (Yue et al., 2017) can also participate in the process of skeletal muscle development. In sheep skeletal muscle development, miR-192 could enhance muscle satellite cell proliferation but inhibit their differentiation *via* targeting RB1 (Zhao et al., 2016). miR-17-92 could promote C2C12 myoblast proliferation and inhibit their differentiation by targeting the ENH1/Id1 signaling axis (Qiu et al., 2016). miR-30-5p could inhibit C2C12 cell differentiation *via* targeting muscleblind-like protein (MBNL) (Zhang et al., 2016). miR-660 could inhibit myogenic differentiation of C2C12 myoblasts by reducing the expression level of ARHGEF12 (Yue et al., 2017). Clearly, miRNAs could play critical roles in skeletal muscle development. However, there still exist many miRNA functions worth being explored and investigated in a future study.

Previous research has shown that miR-452 plays different regulatory roles in various tumors. In hepatocellular carcinoma cells and a breast cancer cell line (MCF-7), miR-452 acts as a tumor-positive miRNA. In hepatocellular carcinoma cells, the expression level of miR-452 is upregulated, then promoting migration (Zheng et al., 2014) and stem-like traits by targeting Sox7 (Zheng et al., 2016). In human breast cancer cell line (MCF-7), miR-452 was downregulated and modulated chemosensitivity of breast cancer cells to Adriamycin (ADR) (Hu et al., 2014). In addition, miR-452 also acts as a tumor-negative miRNA. For example, in non-small-cell lung cancer (NSCLC), miR-452 is downregulated and promotes the invasive capability of NSCLC cells by regulating BMI1 (He et al., 2015). In prostate cancer tissues, miR-452 expression was significantly downregulated and inhibited cancer cell migration and invasion through targeting E3 ubiquitin ligase-1 (WWP1) (Goto et al., 2016). miR-452 was also involved in human osteosarcoma, which suppressed cell proliferation, promoted cell apoptosis, and led to BMI1 protein level decline (Li and Wang, 2016). Moreover, miR-452 had been identified from the skeletal muscle of goat (Wang et al., 2014) and sheep (Zhao et al., 2016) by high-throughput sequencing. However, the function and target genes of miR-452 during C2C12 cell proliferation and differentiation are unknown. In the present study, we found that miR-452 could promote C2C12 cell proliferation and impede their differentiation by targeting *Angiopoietin 1* (*ANGPT1*). The current studies might offer an avenue for exploring the regulation of miR-452 in myogenesis.

## Materials and Methods

### Plasmid Construction

Based on TargetScan and DAVID bioinformatics software analysis, the target gene *ANGPT1* was selected. The wild-type 3’-UTR and the mutant 3′-UTR (the seed region target sequence of miR-452 was changed from GCAAACA to GGTTACA by using overlap PCR) of ANGPT1 were cloned into the psiCheck2 vector between the *Xho*I and *Not*I site. Moreover, the precursor sequence of miR-452 was also cloned into pcDNA3.1(+) vector with the *Hin*dIII and *Bam*HI site. The primer sequences for purpose fragment were listed in Table 1.

**TABLE 1:** Primer Sequences Used for Plasmid Construction.

Name	Primer name	Primer sequence (5′–3′)
miR-452	miR-452-F	*CCCAAGCTT*CCCAGGAGGGTGGTAAGGATG
	miR-452-R	*CGCGGATCC*TCTACTCACCTACCCTCTCCG
*ANGPT1*	ANGPT1-F	*CCGCTCGAG*TGGAGAAGCCACCAGATGAGA
	ANGPT1-R	*AAGGAAAAAAGCGGCCGC*TCAATCATCCCAG GCAGAGAC
	Mut-ANGPT1^*a*^-F	*CCGCTCGAG*TCTGAAGGTTACAATATGGTCT CCC

*^*a*^ Overlap PCR primer of ANGPT1.*

### Cell Culture

HEK293T cells and C2C12 myoblasts (ATCC, New York, NY, United States) were cultured in growth medium with Dulbecco’s modified Eagle’s medium (DMEM) (Gibco) containing 10% fetal bovine serum (FBS) (Transgene) and 1% penicillin-streptomycin (Hyclone) at 37°C with 5% CO_2_. After C2C12 myoblast confluence, the growth medium was replaced by differentiation medium (DM) with DMEM supplemented with 2% horse serum (Solarbio) and 1% penicillin-streptomycin.

### Cell Transfection

In order to explore the effect of miR-452 and its target gene on skeletal myogenesis, pcDNA3.1(+)-miR-452, miR-452 mimics (GenePharma, China), miR-452 inhibitor, and siRNA (Table 2) were transfected into C2C12 myoblasts with Lipofectamine 2000 (Invitrogen), respectively. For proliferation experiments, cell transfection was performed when the cell density is up to 40∼50% with the Opti-MEM medium. After 6 h of transfection, the medium was changed into fresh growth medium (GM) for 24 and 48 h. In the differentiation experiments, transfection was performed when the cell density is up to70–80%. After 6 h of transfection, the cell medium needs to be replaced by DM.

**TABLE 2:** Sequences Information of RNA Oligonucleotides.

Name	Sequences name	Sequences (5′–3′)
	MIMICS	UGUUUGCAGAGGAAACUGAGAC
		CUCAGUUUCCUCUGCAAACAUU
miR-452	Negative control	UUUUCCGAACGUGUCACGUTT
		ACGUGACACGUUCGGAGAATT
	Inhibitors	GUCUCAGUUUCCUCUGCAAACA
	Anti-negative control	CAGUACUUUUGUGUAGUACAA

### Real-Time Quantitative PCR

Total RNA was extracted from C2C12 myoblasts using TRIzol reagent (TakaRa, Japan). A total of 1 μg RNA was reverse transcribed into cDNA by using PrimeScript RT Kit (Takara). For RNA quantification, SYBR Mix (Takara) was performed using the ABI Step One Plus real-time PCR system (Applied Biosystems) following the manufacturer’s instructions. Real-time quantitative PCR (RT-qPCR) parameters were as follows: 30 s at 95°C, 40 cycles of 95°C for 10 s, and 60°C for 1 min, then Melt Curve Stage. The results were normalized by the 18S and β-actin expressions through Ct 2^–△^^△^^*Ct*^ value. The primers for RT-qPCR were listed in Table 3.

**TABLE 3:** Primer Information of miRNA and mRNA Quantitative Reverse Transcription.

	Primer name	Primer sequence (5′–3′)
	Stem-loop RT-miR-452^*a*^	GTCGTATCCAGTGCAGGGTCCGAGGTAT TCGCACTGGATACGACGTCTCAGT
miR-452	miR-452-F	CGCGCGTGT TTGCAGAGGAAACT
	miR-452-R	GTGCAGGGTCCGAGGT
*18s*	18s-F	GTGGTGTTGAGGAAAGCAGACA
	18s-R	TGATCACACGTTCCACCTCATC
*Pax7*	Pax7-F	GCCGCCTTCAACCACCTTCTG
	Pax7-R	GTGTACTGTGCTGCCTCCATCTTG
*CDK1*	CDK1-F	ACGGCTTGGATTTGCTCTC
	CDK1-R	ACGATCTTCCCCTACGACCA
*Myf5*	Myf5-F	CGGATCACGTCTACAGAGCC
	Myf5-R	GCAGGAGTGATCATCGGGAG
*MyoG*	MyoG-F	TGGAGCTGTATGAGACATCCC
	MyoG-R	TGCACAATGCTCAGGGGTCCC
*Mef2c*	Mef2c-F	TGCTGTGCGACTGTGAGATTGC
	Mef2c-R	CTCGTGCGGCTCGTTGTACTC
*ANGPT1*	ANGPT1-F	CTCACAGTACGACAGATTCCACATAGG
	ANGPT1-R	CGAACCACCAACCTCCTGTTAGC
β*-actin*	β-actin-F	GGCACCACACCTTCTACAATG
	β-actin-R	GGGGTGTTGAAGGTCTCAAAC

*^*a*^ Stem-loop RT-miR-452 was used to reverse transcription of miR-452.*

### Western Blot

Cell proteins were extracted from radioimmunoprecipitation assay (RIPA) buffer, and the protein concentration was evaluated with the bicinchoninic acid (BCA) protein assay kit (Solarbio). Sodium dodecylsulfate–polyacrylamide gel electrophoresis (SDS-PAGE) was performed to separate protein, and then the proteins were transferred onto the polyvinylidene fluoride (PVDF) membrane. Firstly, the PVDF membrane was sealed with 5% skim milk powder for 1 h. Secondly, the PVDF membrane was incubated overnight at 4°C with primary antibodies against Myf5 (Abcam; 1:10,000 dilution), MyoG (Abscience; 1:1,000 dilution), Mef2c (Abscience; 1:1,000 dilution), ANGPT1 (Abscience; 1:1,000 dilution), or β-tubulin (Abscience; 1:1,000 dilution). Lastly, the PVDF membrane was incubated with goat anti-immune rabbit IgG (ZSGB-BIO, China; 1:5,000 dilution). The proteins were detected by enhanced chemiluminescence (ECL) reagents (willget Biotech, Shanghai). Images acquisition was performed on the FluorChemMsystem (ProteinSimple) and analyzed using Image-Pro plus software.

### Cell Proliferation Assay

The proliferation state of C2C12 myoblasts was detected by Cell Counting Kit (CCK)-8 and 5-ethynyl-2-deoxyuridine (EdU) in 24-well plate. First, the cultured C2C12 cells in GM were collected 24 and 48 h after transfection of pcDNA3.1(+)-miR-452, pcDNA3.1(+), miR-452 mimics, negative control (NC), miR-452 inhibitors, and anti-negative control (anti-NC). After being washed with PBS, these cells were incubated with CCK-8 reagent (EnoGene, China) for 1 h at 37°C. Then, all treatment group cells were detected by 450 nm absorbance using microplate reader (Thermo Fisher Scientific, United States). To detect the S phase cell number, the EdU reagent (Ribobio, China) was used to perform this. Briefly, the C2C12 cells were transfected, which were cultured in GM for 24 h with medium containing 10 μM EdU before immunostaining. Images were collected using a fluorescence microscope (Nikon, Japan). ImageJ software was used to calculate the number of EdU-positive cells and Hoechst-stained cells; the ratio of EdU-positive cells was calculated with (EdU-positive cells/Hoechst-stained cells) × 100%.

### Dual-Luciferase Reporter Assay

The wild-type or mutant 3′-UTR dual-luciferase reporter vector and the miR-452 mimics or the NC were co-transfected into the HEK293T cells with Lipofectamine 2000. After transfection for 48 h, the luciferase activity analysis was performed by dual-luciferase reporter assay kit. Cells were cracked with 100 μl cell lysis buffer (CLB) per well, and then these plates of cells were collected, after that, 20 μl of the cell lysis supernatant was moved to 96-well plates in triplicate containing 30 μl luciferase reaction reagent per well for firefly luciferase activities, then 30 μl luciferase reaction reagent II was added into the 96-well plates per well for the Renilla luciferase activity assay. The Renilla luciferase activity was normalized to the firefly luciferase activity.

### Statistical Analysis

All experiments were performed for three replicates. All experiment data in this study were presented as mean ± standard error (SE). One-way analysis of variance (ANOVA) and *T*-test were used for statistical analysis with software SPSS21. The value of *P* < 0.05 is considered significantly different, and *P* ≤ 0.01 is considered extremely significantly different.

## Results

### MiR-452 Accelerates C2C12 Myoblast Proliferation

**FIGURE 1:**
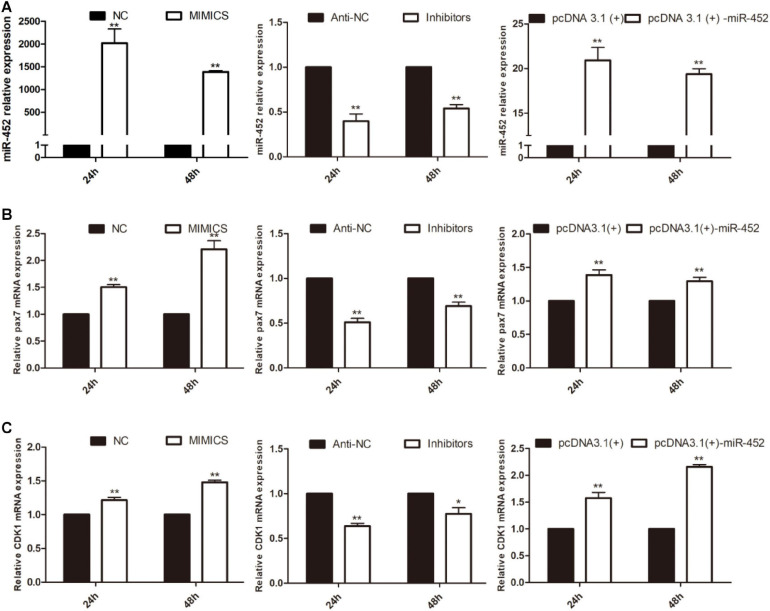
miR-452 accelerates C2C12 cell proliferation. **(A)** miR-452 expression at 24 and 48 h after transfection with miR-452 mimics, negative control (NC), miR-452 inhibitors anti-negative control (anti-NC), pcDNA3.1(+)-miR-452, and pcDNA3.1(+) determined by real-time quantitative PCR in GM. **(B)**. Pax7 mRNA expression at 24 and 48 h after transfection miR-452 mimics, NC, miR-452 inhibitors anti-NC, pcDNA3.1(+)-miR-452, and pcDNA3.1(+) determined by the real-time quantitative PCR. **(C)** CDK1 mRNA expression at 24 and 48 h after transfection miR-452 mimics, NC, miR-452 inhibitors anti-NC, pcDNA3.1(+)-miR-452, and pcDNA3.1(+) determined by the real-time quantitative PCR. Asterisks indicate significant differences. **P < 0.01; *P < 0.01. Data are SEM (mean ± standard error of the mean) of three independent experiments.

**FIGURE 2:**
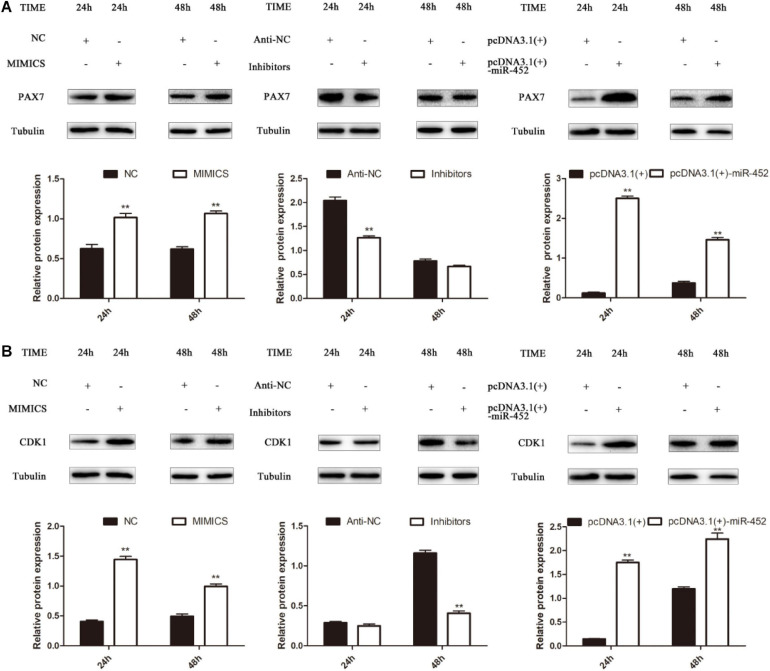
miR-452 accelerates C2C12 cell proliferation. **(A)** Pax7 protein expression at 24 and 48 h after transfection with miR-452 mimics, negative control (NC), miR-452 inhibitors, anti-negative control (anti-NC), pcDNA3.1(+)-miR-452, and pcDNA3.1(+). **(B)** CDK1 protein expression at 24 and 48 h after transfection with miR-452 mimics, negative control (NC), miR-452 inhibitors, anti-negative control (anti-NC), pcDNA3.1(+)-miR-452, and pcDNA3.1(+). Data are represented as M ± SEM. **P < 0.01.

**FIGURE 3:**
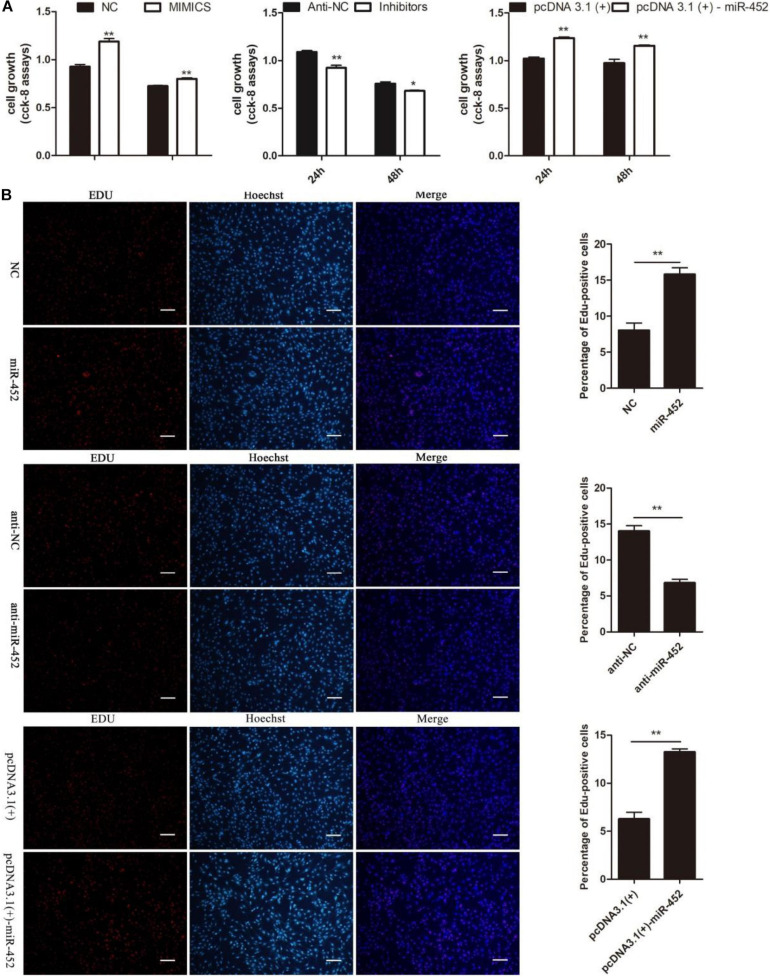
miR-452 accelerates C2C12 cell proliferation. **(A)** The survival of C2C12 cells by using Cell Counting Kit (CCK)-8 assay at 24 h, 48 h after transfection miR-452 mimics, negative control (NC), miR-452 inhibitors, anti-negative control (anti-NC), pcDNA3.1(+)-miR-452, and pcDNA3.1(+). (*n* = 4). **(B)** Representative photo images of 5-ethynyl-2-deoxyuridine (EdU) assay of C2C12 after transfection miR-452 mimics, negative control (NC), miR-452 inhibitors, and anti-negative control (anti-NC) (left) and quantification of EdU-positive cells (right) (n = 4). Bars, 200 μm. Data are represented as M ± SEM. **P < 0.01, *P < 0.05.

To verify the function of miR-452 for C2C12 myoblast proliferation. After function gain or loss of miR-452 in C2C12, we explored the mRNA expression level of *Pax7* and *CDK1* by the RT-qPCR analysis. The results indicated that overexpression of miR-452 promoted the expression of *Pax7* and *CDK1*, whereas inhibition of miR-452 depressed the expression of *Pax7* and *CDK1* (Figures 1A–C). Meanwhile, we also detected the protein level of Pax7 and CDK1 by Western blot analysis. The results showed that the protein expression trend of Pax7 and CDK1 was in accordance with the trend of mRNA expression level (Figures 2A,B). Furthermore, we also used EdU and CCK-8 proliferation assay kit to detect the C2C12 myoblast proliferation index, and the results indicated that overexpression of miR-452 promoted C2C12 myoblast proliferation after CCK-8 assay (Figure 3A) and increased the proportion of EdU-positive cells (Figure 3B). However, inhibition of miR-452 presented an opposite tendency. Taken together, aforementioned results demonstrated that miR-452 promoted C2C12 myoblast proliferation.

### MiR-452 Inhibits C2C12 Myoblast Differentiation

**FIGURE 4:**
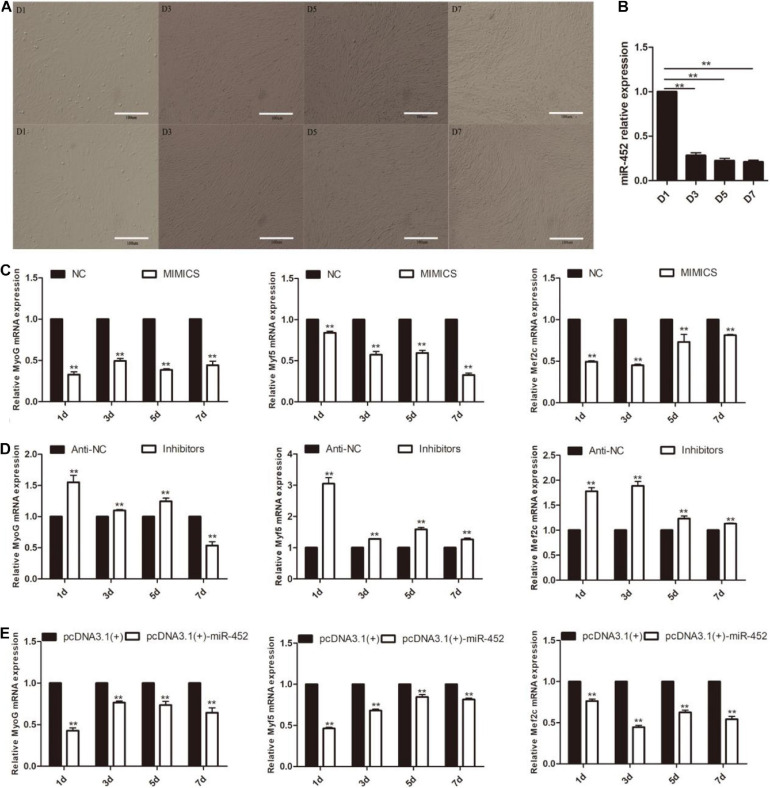
miR-452 inhibits C2C12 cell myogenic differentiation. **(A)** Microscopic images of C2C12 cells after transfection with miR-452 mimics (up) and inhibitors (down) cultured in DM for 1 (D1), 3 (D3), 5 (D5), and 7 (D7) days. Bars, 100 μm. **(B)** miR-452 expression was determined by the real-time quantitative PCR in differentiated C2C12 myoblasts on various days (D1, D3, D5, and D7). **(C–E)** MyoG, Myf5, Mef2c mRNA expression on D1, D3, D5, D7 after transfection with miR-452 mimics, negative control (NC), miR-452 inhibitors, anti-negative control (anti-NC), pcDNA3.1(+)-miR-452, and pcDNA3.1(+) determined by real-time quantitative PCR in DM. Data are represented as M ± SEM. **P < 0.01.

After C2C12 myoblast confluence, the growth medium was replaced by differentiation medium to induce myoblast differentiation. We detected miR-452 expression level during C2C12 myoblast differentiation stage on days 1, 3, 5, and 7. The results showed that miR-452 expression levels presented a decreased trend (Figures 4A,B). Obviously, the expression level of miR-452 was higher at the early stage of C2C12 myoblast differentiation than in the other differentiation period. So, we speculated that miR-452 might possess a function for determining C2C12 myoblast differentiation. To further elucidate the function of miR-452 for C2C12 myoblast differentiation, the myogenic marker genes like Myf5, MyoG, and Mef2c mRNA and protein expression levels were detected.

**FIGURE 5:**
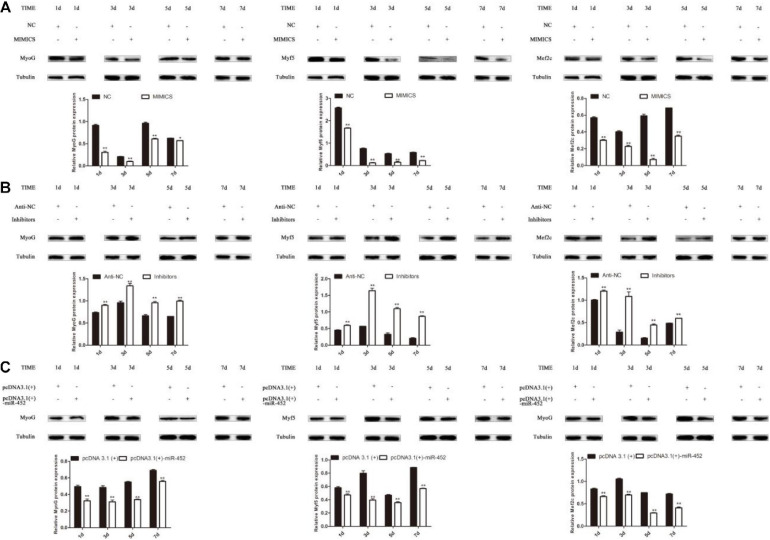
miR-452 inhibits C2C12 cell myogenic differentiation. **(A–C)** MyoG, Myf5, Mef2c protein expression on D1, D3, D5, D7 after transfection with miR-452 mimics, negative control (NC), miR-452 inhibitors and anti-negative control (anti-NC), pcDNA3.1(+)-miR-452, and pcDNA3.1(+) in DM. Data are represented as M ± SEM. **P < 0.01, *P < 0.05.

The results show that overexpression of miR-452 could reduce these marker genes mRNA (Figures 4C,E) and protein level (Figures 5A,C). On the contrary, inhibition of endogenous miR-452 could enhance the mRNA (Figure 4D) and protein expression level (Figure 5B) of these myogenic marker genes. All these results demonstrated that miR-452 could impede C2C12 myoblast myogenic differentiation.

### MiR-452 Directly Targets 3′-UTR of ANGPT1

**FIGURE 6:**
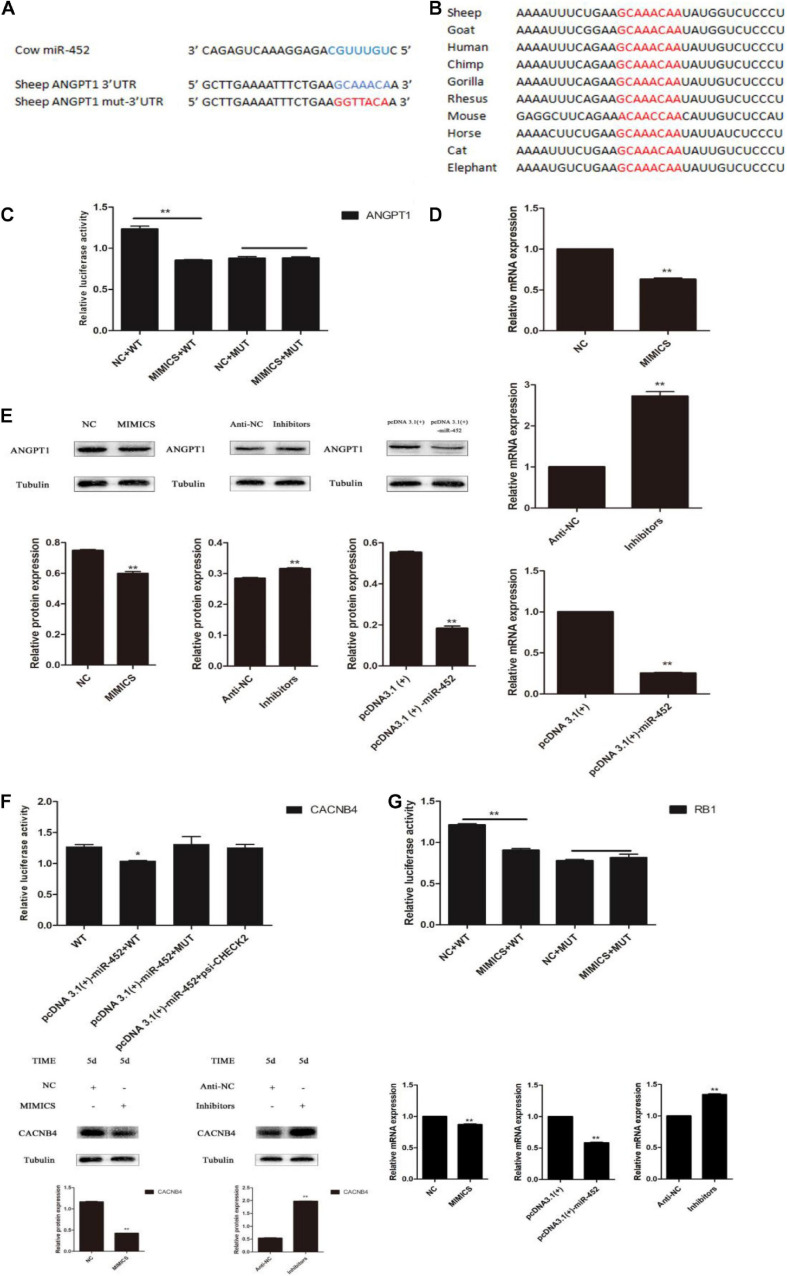
miR-452 directly targets 3′ UTR of ANGPT1. **(A)** The predicted binding site (blue) and mutated site (red) of miR-452 in the 3′-UTR of sheep ANGPT1. **(B)** The conservation of the miR-452 binding site (red) in the 3′-UTR of ANGPT1 from 10 different species. **(C)** Dual-luciferase activity assay of the wild-type (WT) or mutant (MUT) 3′-UTR of ANGPT1. miR-452 mimics or negative control (NC) were co-transfected with the WT or MUT 3′-UTR luciferase reporters of ANGPT1 in HEK293T cells. **(D)** ANGPT1 mRNA expression after transfection with miR-452 mimics, NC, miR-452 inhibitors, anti-negative control (anti-NC), pcDNA3.1(+)-miR-452, and pcDNA3.1(+) in C2C12 myoblast differentiation. **(E)** ANGPT1 protein expression after transfection with miR-452 mimics, NC, miR-452 inhibitors, anti-NC, pcDNA3.1(+)-miR-452, and pcDNA3.1(+) in C2C12 myoblast differentiation in DM on D5. β-tubulin was used as an internal control. **(F)** Dual-luciferase activity assay of the WT or MUT 3′-UTR of CACNB4. miR-452 mimics or NC were co-transfected with the wild-type or mutant 3′-UTR luciferase reporters of CACNB4 in HEK293T cells and CACNB4 protein expression after transfection with miR-452 mimics, NC, miR-452 inhibitors, and anti-negative control (anti-NC) in C2C12 myoblast differentiation in DM on D5. **(G)** Dual-luciferase activity assay of the WT or MUT 3′-UTR of RB1. miR-452 mimics or NC were co-transfected with the WT or MUT 3′-UTR luciferase reporters of RB1 in HEK293T cells and RB1 mRNA expression after transfection with miR-452 mimics, NC, miR-452 inhibitors, anti-NC, pcDNA3.1(+)-miR-452, and pcDNA3.1(+) in C2C12 myoblast differentiation. Data are represented as M ± SEM. **P < 0.01, *P < 0.05.

Depending on the regulatory mechanism of miRNAs, we found that sheep gene *ANGPT1* 3′-UTR contained a miR-452 seed region binding site (Figures 6A,B) through bioinformatics analysis. To further valuate the target relationship between miR-452 and these genes, we cloned and inserted the wild-type or mutant 3′UTR sequence of these genes into psi-Check2 vector (Figure 6C). The wild-type reporter vector was co-transfected with miR-452 mimic or NC into HEK293T cells. The results indicated that the ratio of Renilla/Firefly decreased significantly in the miR-452 mimic group. Meanwhile, the mutant-type reporter vector was co-transfected with miR-452 mimic or NC into HEK293 cell. However, there is no significant luciferase activity change in miR-452 mimic group compared to the control group (Figures 6C,F,G). Moreover, overexpression of miR-452 reduced the expression level of *ANGPT1* mRNA and protein level in C2C12 myoblasts (Figures 6D,E). But, inhibition of endogenous miR-452 induced the expression level of *ANGPT1* mRNA and protein levels in C2C12 myoblasts (Figures 6D,E). Therefore, all these results demonstrated that *ANGPT1* is a direct target gene of miR-452.

### Knockdown of ANGPT1 Promotes Myoblast Proliferation and Inhibits Myogenic Differentiation

**FIGURE 7:**
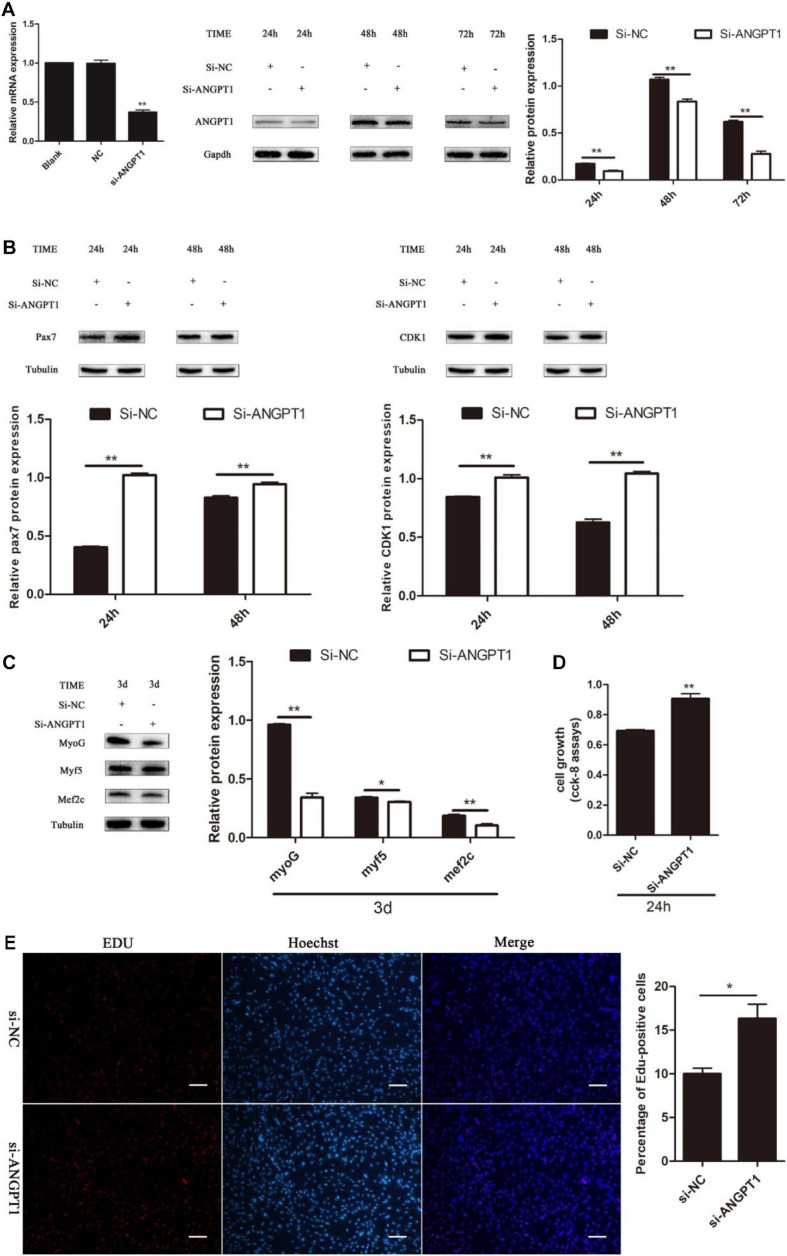
Knockdown of ANGPT1 promotes proliferation as well as inhibits myogenic differentiation of C2C12. **(A)** The mRNA expression level of the si-ANGPT1 at 36 h, and the protein expression level of the si-ANGPT1 at 24, 48, 72 h after transfection with siRNA against mouse ANGPT1 (si-ANGPT1) or siRNA control (si-NC). Quantification of si-ANGPT1 was normalized to glyceraldehyde-3-phosphate dehydrogenase (GAPDH) (*n* = 3). **(B)** The protein expression level of Pax7 and CDK1 after transfection with si-ANGPT1 or si-NC. Quantification of Pax7 and CDK1 was normalized to β-tubulin (*n* = 3). **(C)** The protein expression level of MyoG, Myf5, and Mef2c after transfection with si-ANGPT1 or si-NC. Quantification of MyoG, Myf5, and Mef2c was normalized to β-tubulin (*n* = 3). **(D)** The proliferation of C2C12 cells by Cell Counting Kit (CCK)-8 assay at 24 h after transfection with si-ANGPT1 or si-NC (*n* = 6). **(E)** Representative photo images of 5-ethynyl-2-deoxyuridine (EdU) assay of C2C12 after transfection with si-ANGPT1 or si-NC (left) and quantification of EdU-positive cells (right) (*n* = 4). Bars, 200 μm. The results are shown as the mean ± SEM. *P < 0.05; **P < 0.01. In this experiment, paired two-tailed *t*-test was performed to calculate *p*-value, unpaired two-tailed *t*-test was performed to calculate *p*-value.

The regulatory role of *ANGPT1* on the proliferation and differentiation of C2C12 myoblasts is unclear. To verify the function of *ANGPT1* for C2C12 myoblast proliferation and differentiation, small-interfering RNA of *ANGPT1* was used to knockdown its expression level. The results indicated that the mRNA and protein expression levels of *ANGPT1* were significantly reduced (Figure 7A). After loss of *ANGPT1*, during C2C12 myoblast proliferation stage, the Pax7 and CDK1 protein expression levels were upregulated (Figure 7B). However, during C2C12 myoblast differentiation stage, the MyoG, Myf5, and Mef2c protein expression level were downregulated (Figure 7C). In addition, the C2C12 myoblast proliferation status was detected by using CCK-8 reagent. The results revealed that knockdown of *ANGPT1* promoted myoblast proliferation (Figures 7D,E). Meanwhile, the number of EdU-positive cells was also detected by using EdU reagent. The results showed that the proportion of EdU-positive cells increased after transfection with si-ANGPT1 (Figures 7D,E). Altogether, these results indicated that knockdown of *ANGPT1* could significantly accelerate C2C12 myoblast proliferation but inhibit myogenic differentiation.

## Discussion

Skeletal muscle myogenesis is a complex process, which has been demonstrated to be influenced by many factors likely MRFs and MEF2. Besides these regulatory factors, some non-coding RNA has been reported to be involved in myogenesis, such as miRNAs. In this study, we found that miR-452 could promote C2C12 myoblast proliferation and impede their differentiation *via* targeting *ANGPT1*.

At the molecular level, during C2C12 myoblast proliferation stage, the mRNA and protein expression levels of *Pax7* and *CDK1* were detected at 24 and 48 h. Both genes are involved in myoblast proliferation. *Pax7* had been reported to be involved in myogenic satellite cell maintenance and regenerative capacity (Padilla-Benavides et al., 2015). *CDK1* had been demonstrated to affect Myod half-life and myogenic activity (Kitzmann et al., 1999). Our current results showed that overexpression of miR-452 significantly increased the expression level of these genes. But inhibition of miR-452 got an opposite result. During C2C12 myoblast differentiation stage, the MRFs Myf5, MyoG, and Mef2c mRNA and protein expression levels were detected on days 1, 3, 5, and 7. The results had shown that overexpression of miR-452 significantly reduced the mRNA and protein expression of these genes and *vice versa*.

In mammals, miRNAs affect the stability of mRNA or impede the protein translation process by targeting the 3′ UTR of mRNA (Stark et al., 2005). Through Targets can, DAVID software analysis, and dual-luciferase reporter assay, we found that*ANGPT1* was the target gene of miR-452. *ANGPT1* encodes secretory glycoproteins, which belongs to the angiopoietin family. All angiopoietin activates endothelial specific tyrosine kinase receptor (Tie-2) through phosphorylation, thereby playing an important role in vascular development and angiogenesis (Suri et al., 1996; Yancopoulos et al., 2000). In particular, *ANGPT1* can induce proliferation of endothelial cells (Abdelmalak et al., 2008). Moreover, *ANGPT1* had been demonstrated to enhance skeletal muscle regeneration in mice by inducing blood vessel formation (Mofarrahi et al., 2015). In this research, we found that loss of *ANGPT1* could promote C2C12 myoblast proliferation and inhibit myogenic differentiation.

In summary, the results of the current study indicated that miR-452 promoted C2C12 myoblast proliferation and inhibited their myogenic differentiation. And loss of *ANGPT1* presented a similar result, which are consistent with after overexpression of miR-452. Combining with dual-luciferase reporter assay and a series of evidence suggested that *ANGPT1* was a target gene of miR-452 in C2C12 myoblasts.

## Data Availability Statement

The original contributions presented in the study are included in the article/supplementary material, further inquiries can be directed to the corresponding author/s.

## Author Contributions

XF, CZ, and LY made substantial contributions to the conception and design of the experiment. LY, QQ, JW, and CS made acquisition, analysis, and interpretation of data. LY, QQ, and JW were involved in drafting the manuscript. YW, XC, HC, and LH performed the experiments and revised the draft critically. XF and CZ gave final approval of the version to be published and agreed to be accountable for all aspects of the work in ensuring that questions related to the accuracy or integrity of any part of the work are appropriately investigated and resolved. All authors had participated sufficiently in the work to take public responsibility for appropriate portions of the content. All authors contributed to the article and approved the submitted version.

## Conflict of Interest

The authors declare that the research was conducted in the absence of any commercial or financial relationships that could be construed as a potential conflict of interest.
